# Reprogramming the murine colon cancer microenvironment using lentivectors encoding shRNA against IL-10 as a component of a potent DC-based chemoimmunotherapy

**DOI:** 10.1186/s13046-018-0799-y

**Published:** 2018-06-28

**Authors:** Joanna Rossowska, Natalia Anger, Agnieszka Szczygieł, Jagoda Mierzejewska, Elżbieta Pajtasz-Piasecka

**Affiliations:** Hirszfeld Institute of Immunology and Experimental Therapy, Polish Academy of Sciences, ul. R. Weigla 12, 53-114 Wroclaw, Poland

**Keywords:** Dendritic cells, Interleukin 10, Lentivectors, Cyclophosphamide, Colon carcinoma, MC38, Immunotherapy, IL-10 blocking

## Abstract

**Background:**

The excessive amounts of immunosuppressive factors present in a tumor microenvironment (TME) reduce the effectiveness of cancer vaccines. The main objective of our research was to improve the effectiveness of dendritic cell (DC)-based immunotherapy or chemoimmunotherapy composed of cyclophosphamide (CY) and DCs by application of lentivectors encoding shRNA specific to IL-10 (shIL10 LVs) in murine colon carcinoma MC38 model.

**Methods:**

The efficacy of shIL10 LVs in silencing of IL-10 expression was measured both in vitro and in vivo using Real-Time PCR and ELISA assays. In addition, the influence of intratumorally inoculated lentivectors on MC38 tumor microenvironment was examined using flow cytometry method. The effect of applied therapeutic schemes was determined by measurement of tumor growth inhibition and activation state of local and systemic immune response.

**Results:**

We observed that intratumorally inoculated shIL10 LVs transduced tumor and TME-infiltrating cells and reduced the secretion of IL-10. Application of shIL10 LVs for three consecutive weeks initiated tumor growth inhibition, whereas treatment with shIL10 LVs and BMDC/TAg did not enhance the antitumor effect. However, when pretreatment with CY was introduced to the proposed scheme, we noticed high MC38 tumor growth inhibition accompanied by reduction of MDSCs and Tregs in TME, as well as activation of potent local and systemic Th1-type antitumor response.

**Conclusions:**

The obtained data shows that remodeling of TME by shIL10 LVs and CY enhances DC activity and supports them during regeneration and actuation of a potent antitumor response. Therefore, therapeutic strategies aimed at local IL-10 elimination using lentiviral vectors should be further investigated in context of combined chemoimmunotherapies.

**Electronic supplementary material:**

The online version of this article (10.1186/s13046-018-0799-y) contains supplementary material, which is available to authorized users.

## Background

Novel immunotherapeutic strategies, including cancer vaccines can successfully promote renewal and activation of long-term antitumor immune response [1–4]. However, excessive amounts of soluble factors secreted in a tumor microenvironment (TME) e.g. TGF-β, IL-10 or VEGF reduce the effectiveness of the applied treatment and cause further tumor progression [5–7]. Therefore, it is within reason to eliminate suppressor cytokines prior to application of cancer vaccines. In our latest research we focused our attention on reduction of the negative impact of IL-10 in TME on antitumor response. IL-10 is an immunomodulatory cytokine, which in normal conditions prevents tissue damaging by inflammatory cytokines. However, during tumor development, its prolonged production by tumor infiltrating cells and certain types of cancer cells induces immunosuppression [8, 9]. It was shown that IL-10 secreted in TME causes changes in classical and nonclassical MHC I molecule expression on the tumor cell surface and thereby inhibits the cytolytic activity of CTLs and NK cells towards tumor cells [10, 11]. Its effect on monocytes and dendritic cells (DCs) is also well-documented. IL-10 disrupts the process of antigen presentation and inhibits proinflammatory cytokine secretion by antigen presenting cells (APCs), thus inducing immune tolerance [12]. Apart from high immunosuppressive activity, the elevated level of IL-10 leads to increased proliferation and chemoresistance of malignant cells, which is often accompanied by their escape from apoptosis [9, 13].

In order to address the aforementioned drawbacks, elimination of IL-10 was proposed as a potent strategy in fighting cancer. In an attempt to enhance the antitumor immune response, various strategies aiming to neutralize or revert the effects of IL-10 i.e., antibodies, immunoadhesins, antisense oligodeoxynucleotides or low dose of cyclophosphamide [14–18], are developed. In our study we decided to decrease the production of IL-10 in TME using lentivectors encoding shRNA that targets murine IL-10 (shIL10 LVs). The lentivectors were produced using the third generation lentiviral system, which is characterized by less mutagenic activity in comparison to traditional retroviral vectors and increased safety provided by integrated self-inactivating promoters [19, 20]. The shIL10 LVs were applied as components of a DC-based immunotherapy or chemoimmunotherapy, which consisted of an immunomodulatory dose of cyclophosphamide (CY) and DCs. The main objective of our work was to improve the antitumor effect of DCs stimulated with tumor antigens by diminishing the IL-10 production in TME. The gathered data indicates that intratumoral injections of shIL10 LVs decreased the production of IL-10 in tumor, while application of shIL10 LVs with DC-based vaccines, especially after pretreatment with CY, induced a significant MC38 tumor growth delay accompanied by reduced numbers of MDSCs and Tregs in TME, as well as enhanced local and systemic Th1-type antitumor activity.

## Methods

### Mice

Female C57BL/6 mice were obtained from the Center for Experimental Medicine of the Medical University of Bialystok (Bialystok, Poland). All experiments were performed in accordance with EU Directive 2010/63/EU for animal experiments and were approved by the 1st Local Ethics Committee for Experiments with the Use of Laboratory Animals, Wroclaw, Poland (authorization number 11/2015). After the experiments, the mice were sacrificed by cervical dislocation.

### Cell culture

The in vivo growing cell line of MC38 murine colon carcinoma from the Tumor Bank of the TNO Radiobiology Institute, Rijswijk, Holland, was adapted to in vitro conditions as described by Pajtasz-Piasecka et al. [21]. The cell culture was maintained in RPMI 1640 (Gibco) supplemented with 100 U/ml penicillin (Polfa), 100 mg/ml streptomycin (Polfa), 0.5% sodium pyruvate (Sigma-Aldrich), 2-mercaptoethanol (Sigma-Aldrich) and 5% of fetal bovine serum (FBS, Sigma-Aldrich). The Lenti-X™ 293 T cell line (Clontech) was maintained in high-glucose Dulbecco’s Modified Eagle Medium (Gibco) supplemented with 100 U/ml penicillin, 100 mg/ml streptomycin, 0,5% sodium pyruvate and 10% of FBS. BMDCs were generated from bone marrow of healthy C57BL/6 mice according to the protocol described in our previous publication [22]. The cells were cultured in RPMI supplemented with 10% FBS in presence of rmGM-CSF (ImmunoTools, 40 ng/ml) and rmIL-4 (ImmunoTools, 10 ng/ml). After 6 days the loosely attached immature dendritic cells were used for further tests or stimulated with tumor antigens and applied to mice as anti-tumor vaccines.

### Lentiviral vector production

**Fig. 1:**
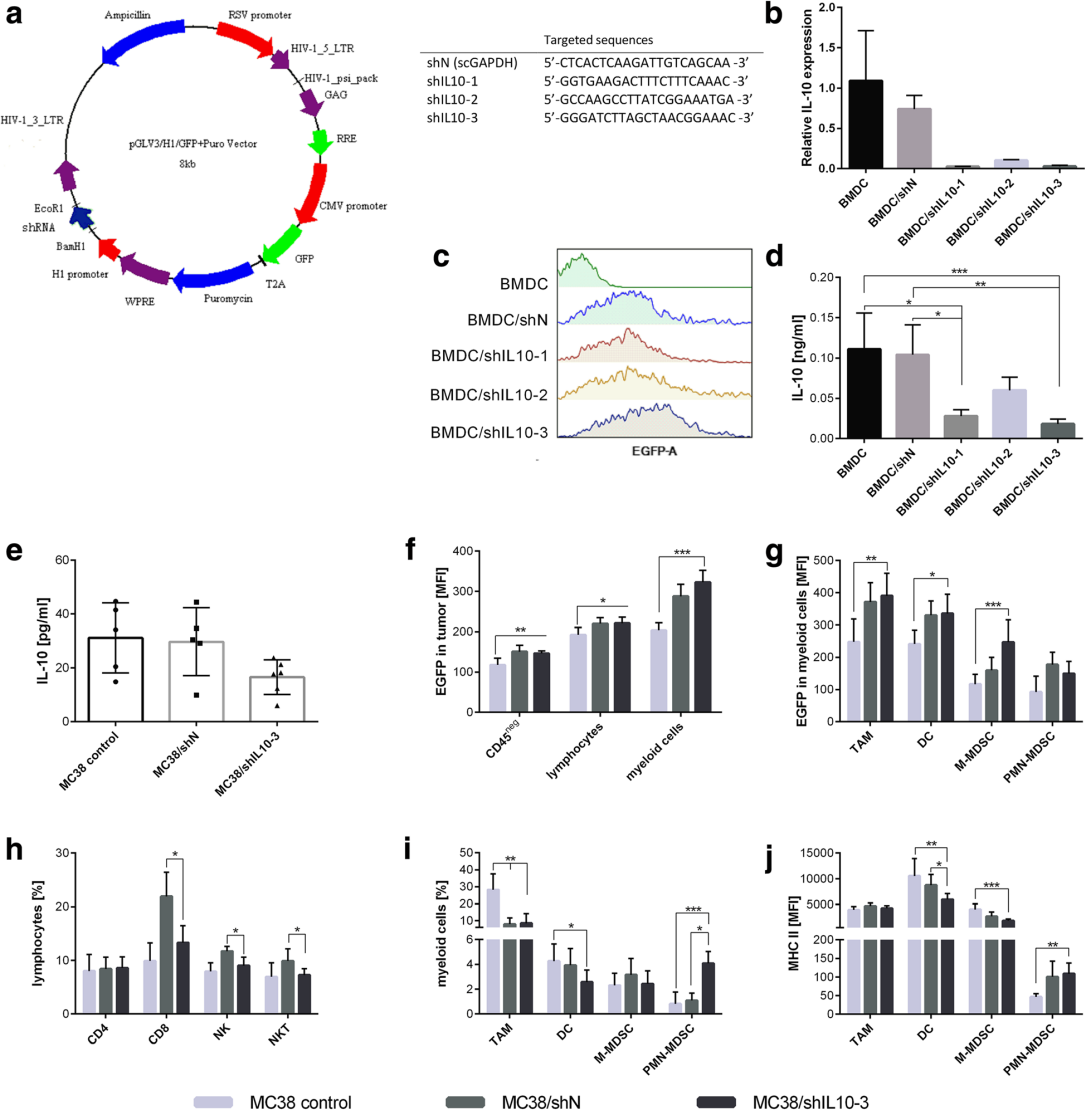
Effectiveness of IL-10 silencing in vitro and in vivo. **a** Scheme of lentiviral vector encoding shRNA against murine IL-10 or scrambled sequence against GAPDH (shN) and selected shRNA sequences used in experiments; **b** Relative expression of IL-10 mRNA in transduced BMDC measured using Real-Time PCR; **c** EGFP expression in BMDC on the 2nd day after transduction with LVs as a control of the transduction effectiveness; **d** Concentration of IL-10 in supernatants collected after 24 h culture of shIL10 transduced and control BMDC measured using ELISA. The results are given as a mean ± SD calculated for three repeats in three independent experiments; **e-j** Intratumoral activity of shIL10–3 LVs. MC38 tumors were dissected on the 6th day after triple intratumoral injection of LVs encoding shIL10–3 or shN; **e** IL-10 produced by cells isolated from tumors; **f** Differences in EGFP expression between tumor cells, and lymphocytes or myeloid cells infiltrating tumor; **g** EGFP expression in defined subpopulations of myeloid cells; **h, i** The percentage of selected lymphocytic or myeloid cell subpopulations among leukocytes infiltrating into tumor after LVs injection; **j** MHC II expression in defined subpopulations of myeloid cells. To calculate the mean ± SD, at least six mice per group were analyzed. The differences between the groups were estimated using non-parametric Kruskal-Wallis test followed by Dunn’s multi comparison test (* *p* < 0.05, ** *p* < 0.01, *** *p* < 0.001)

Lentiviral vectors (LVs) were produced using the third generation lentiviral system consisting of pMDLg/pRRE, pRSV-Rev, pMD2.G (the plasmids were a gift from Didier Trono; Addgene plasmids # 12251, 12253, 12259) and expression plasmids pGLV-H1-GFP + Puro (EzBiolab). The expression plasmids encoded three different shRNA sequences against IL-10, EGFP and puro^r^ genes as selectable markers (Fig. 1). The control vector encoded a scrambled sequence of shRNA against human GAPDH (shN). LVs encoding sequences of shRNA were produced and concentrated as described in our previous publication [23]. Briefly, Lenti-X cells (ClonTech) were co-transfected with vectors and cultured for 48 h. The LVs-containing supernatant was collected and concentrated by PEG 6000 (Sigma-Aldrich) precipitation. The titer of the LVs was determined by serial dilution using MC38 cells.

### Determination of IL-10 silencing efficiency

BMDCs were transduced with lentiviral vectors encoding shRNA that silence IL-10. For reference, cells transduced with vectors encoding scrambled shRNA against human GAPDH, as well as untransduced cells were used. After three days, the efficiency of transduction was determined by flow cytometry (LSR Fortessa, Becton Dickinson) as percentage of EGFP^+^ cells. The expression of IL-10 was measured by Real-Time PCR. Total RNA was isolated using NucleoSpin RNA kit (Mecherey-Nagel) and reverse-transcribed with First Strand cDNA Synthesis Kit (ThermoFisher). Real-Time PCR was performed using TaqMan® Universal PCR Master Mix and TaqMan® Gene Expression Assay primers for IL-10 (Applied Biosystem) in reference to HPRT gene. Production of IL-10 by BMDCs was evaluated by the concentration of the cytokine in supernatant from above a 24 h culture (0.5 × 10^6^ cells/ml) by ELISA (eBioscience).

### Intratumoral activity of LVs

Eight to ten-week old female C57BL/6 mice were subcutaneously inoculated in the right flank with MC38/0 cells (1.1 × 10^6^/0.2 ml/mouse). On the 14th, 15th and 17th day of the experiment, mice were injected i.t. with LVs encoding shRNA against IL-10 (shIL10–3, 2x10^6^TU/50 μl/mouse) or reference LVs encoding scrambled shRNA against human GAPDH (shN). Two days after the third injection, the mice were sacrificed and their tumor nodules were dissected and homogenized. Efficacy of transduction in tumors was measured by flow cytometry as the fluorescence intensity of EGFP among cells isolated from tumors. Concentration of IL-10 was estimated by ELISA in supernatants collected from 24 h culture of 5 mg tumor tissue/ml. Myeloid and lymphocyte populations in tumors were analyzed using LSR Fortessa with Diva software (Becton Dickinson) after staining with fluorochrome-conjugated antibodies: CD45 V500, CD3 PE-CF594, CD19 PE-CF594, CD49b PE-CF594 (all from BD Biosciences), CD11b PerCP-Cy5.5, CD11c BV650, F4/80 AlexaFluor 700, Ly6C BV510, Ly6G BV605, MHC II APC-Cy7, for myeloid cell identification (all from Biolegend) and CD45 BV605, CD3 BV650, CD4 FITC, CD8 APC-Fire, (all from BioLegend) for lymphocyte identification.

### Therapeutic treatment schedule

Eight to ten-week old female C57BL/6 mice were s.c. inoculated in the right flank with MC38/0 cells (1.1 × 10^6^/0.2 ml/mouse). On the 12th day of experiment (exp. B), the mice were injected i.p. with cyclophosphamide (CY, Baxter, 150 mg/kg body weight). On the 14th, 21st and 28th day of the experiment (exp. A, exp. B), the mice were injected i.t. with LVs encoding shRNA against IL-10 (shIL10–3 LVs, 2 × 10^6^TU/50 μl/mouse), reference LVs encoding scrambled shRNA against human GAPDH (shN) or concentrated Lenti-X culture medium (PEG). On the 15th, 22nd and 29th day, tumor antigen-stimulated dendritic cell-based vaccines were applied p.t. (BMDC/TAg, 2 × 10^6^/0.2 ml/mouse in exp. A or 0.2 × 10^6^/0.2 ml/mouse in exp. B). Detailed procedure of BMDC antigenic stimulation was described in our previous publication [24]. On the 36th day, one week after the third DC-based vaccination, 6 mice from each group were sacrificed and their spleens and tumor nodules were dissected, homogenized and stored in liquid nitrogen for further analyses. Procedure of tumor growth monitoring was presented by Pajtasz-Piasecka [25]. The therapeutic effect of the treatment was evaluated using TGI (tumor growth inhibition). Statistical differences were calculated using Friedman and Dunn’s multicomparison tests.

### Analysis of antitumor response of effector spleen cells

Spleen cells obtained from control or treated tumor-bearing mice were restimulated during co-culture with mitomycin C-treated MC38 cells (50 mg MitC/3 × 10^6^ cells; 30 min., 37 °C) in presence of recombinant human IL-2 (200 U/ml). After 5 days, the cells and supernatants were collected. Cytotoxic activity of restimulated splc toward DiO-labeled MC38 target cells, as well as the ability of effector cells to secrete lytic granules were measured as previously described [26]. Cytotoxic cells were identified using anti-CD107 APC, anti-CD8 PE-Cy7 and anti-CD49b PE monoclonal antibodies (BioLegend). In order to determine the polarization of systemic immune response followed by applied treatment, Tbet and FoxP3 expression, and IFN-γ production by T cells was measured. Spleen cells, obtained from treated and control mice, were stimulated with ConA (0.5 μg/ml; Sigma) and IL-2 (200 U/ml) for 48 h. Then cells were harvested and after staining with fluorochrome-conjugated antibodies: CD4 FITC and CD8 APC-Fire (BioLegend) were fixed and permeabilized to intracellular staining of Tbet, FoxP3 and IFN-γ with following antibodies: anti-Tbet PE-Cy7, anti-FoxP3 APC, anti-IFN-γ PE (eBioscience). Flow cytometry analyses were performed using FACSFortessa with FACSDiva software (Becton Dickinson).

### Determination of cytokine production

Production of cytokines by BMDC, cells isolated from tumors and restimulated spleen cells was evaluated using commercially available ELISA kits (IL-10, IL-4 - BD Biosciences; IFN-γ - eBioscience) according to the manufacturer’s instructions.

### Analysis of myeloid cells and lymphocytes in tumors of mice after the therapy

Tumor cells isolated from mice were thawed and stained with LIVE/DEAD Fixable Violet Dead Staining Kit (ThermoFisher). Subsequently, the cells were divided and stained with cocktails of fluorochrome-conjugated monoclonal antibodies: CD3 PE-CF594, CD19 PE-CF594, CD49b PE-CF594 (all from BD Biosciences), CD45 BV605, CD11b PerCP-Cy5.5, CD11c BV650, F4/80 AlexaFluor 700, Ly6C PE, Ly6G APC-Cy7, MHC II FITC, CD80 PE-Cy7 (all from Biolegend) for myeloid cell identification and CD45 BV605, CD3 BV650, CD4 FITC, CD8 APC-Fire, CD25 PE, CD44 PE-Cy7, CD62L PerCP-Cy5.5 (all from BioLegend) for lymphocytes identification. Then, the cells were fixed using FoxP3 Fixation Permeabilization Staining Kit (eBioscience). Tumor cells stained with myeloid or lymphocyte cocktail were additionally incubated with anti-CD206 APC (BioLegend) or FoxP3 APC (eBioscience) antibodies, respectively. The analysis was performed using FACSFortessa flow cytometer with Diva software (Becton Dickinson).

### Statistics

All the data was analyzed using GraphPad Prism 6 software. The statistical significance in kinetics of tumor growth was calculated using Friedman test followed by Dunn’s multi comparison post-hoc test. In all remaining analyses the statistical differences were calculated using the nonparametric Kruskal-Wallis test for multiple independent groups followed by Dunn’s multi comparison post-hoc test. Differences with a *p*-value < 0.05 were regarded as significant.

## Results

### In vitro and in vivo activity of shIL10 LVs

In the first step of our study, we estimated the effectiveness of cell transduction of three different murine IL-10 targeting shRNA sequences, which were cloned into third generation lentiviral vectors. Since MC38 tumor cells are not able to produce IL-10, the efficacy of IL-10 expression silencing of the constructed lentivectors was determined using bone marrow-derived dendritic cells (BMDC). Based on RT-PCR and ELISA, as well as flow cytometry data, which were used to monitor the quality of the transduction process, the shIL10–3 sequence was selected for further in vivo experiments (Fig. 1a-d) as it was determined to exhibit the highest potency in IL-10 downregulation. Intratumoral injection of shIL10–3 LVs into MC38 bearing mice confirmed their activity in tumor microenvironment. On the 6th day after we started the triple inoculations of shIL10–3 LVs, the cells isolated from MC38 tumor nodules showed decreased ability to produce IL-10 compared to both untreated and shN-treated controls (Fig. 1e). The analysis of EGFP expression in tumor cells, as well as tumor infiltrating lymphocytes revealed only slight changes in their fluorescence intensity, while EGFP expression in myeloid cells identified in tumors from the group treated with shIL10–3 LVs was significantly higher than that of the control (Fig. 1f). Further analysis of myeloid cell subpopulations confirmed increased expression of EGFP in tumor associated macrophages (TAM), DCs, monocytic myeloid-derived suppressor cells (M-MDSCs) and granulocytic myeloid-derived suppressor cells (PMN-MDSCs). However, there were no differences in EGFP expression between groups treated with shN and shIL10–3 LVs in all-myeloid cell subpopulations with the exception of M-MDSCs (Fig. 1g). Furthermore, we examined the influence of shIL10–3 LVs on changes in the proportions of the tumor infiltrating lymphocytes, as well as myeloid cells. Analysis of lymphocytes, NK and NKT cells showed that shN LVs strongly activated the antiviral response. Interestingly, the virus effect was diminished in mice that received shIL10–3 LVs, where the number of CTLs, NK and NKT cells was not much higher than in the untreated group (Fig. 1h). In addition, the shIL10–3 LVs affected myeloid cell proportions in tumor. IL-10 silencing LVs significantly increased the influx of PMN-MDSCs, as well as reduced the number of DCs in tumor (Fig. 1i). Moreover, DCs and M-MDSCs that remained in the tumor after shIL10–3 LVs inoculation were characterized by lower expression of MHC II compared to the control groups (Fig. 1j). Additionally to answer the question if LV-based transduction was restricted to tumor nodules, we analyzed also EGFP fluorescence intensity in tumor draining lymph nodes (tLNs; Additional file 1: Figure S1). Data showed that EGFP fluorescence in the LVs treated groups slightly increased compared to untreated control, both in tLN-derived myeloid and lymphoid cells. However, the changes in the EGFP intensity in tLNs were considerably lower than those observed in tumor nodules. Moreover, the highest fluorescence was detected in the tLN-derived myeloid cells from shIL10–3 LVs group. This observation could indicate that tLNs were settled by myeloid cells from tumors. Additionally, the migration was more efficient in the group treated with shIL10–3 LVs. There is also possibility that i.t. inoculated LVs spread to tLNs. However, taking into consideration small changes in EGFP fluorescence this phenomenon was limited. In conclusion, the obtained data showed that myeloid cell subpopulations were the main target of intratumorally injected LVs, and in contrast to the shN control, there was no visible antiviral response induction on the 6th day after shIL10–3 LVs inoculation.

### The shIL10 LVs as a component of potent antitumor therapies

**Fig. 2:**
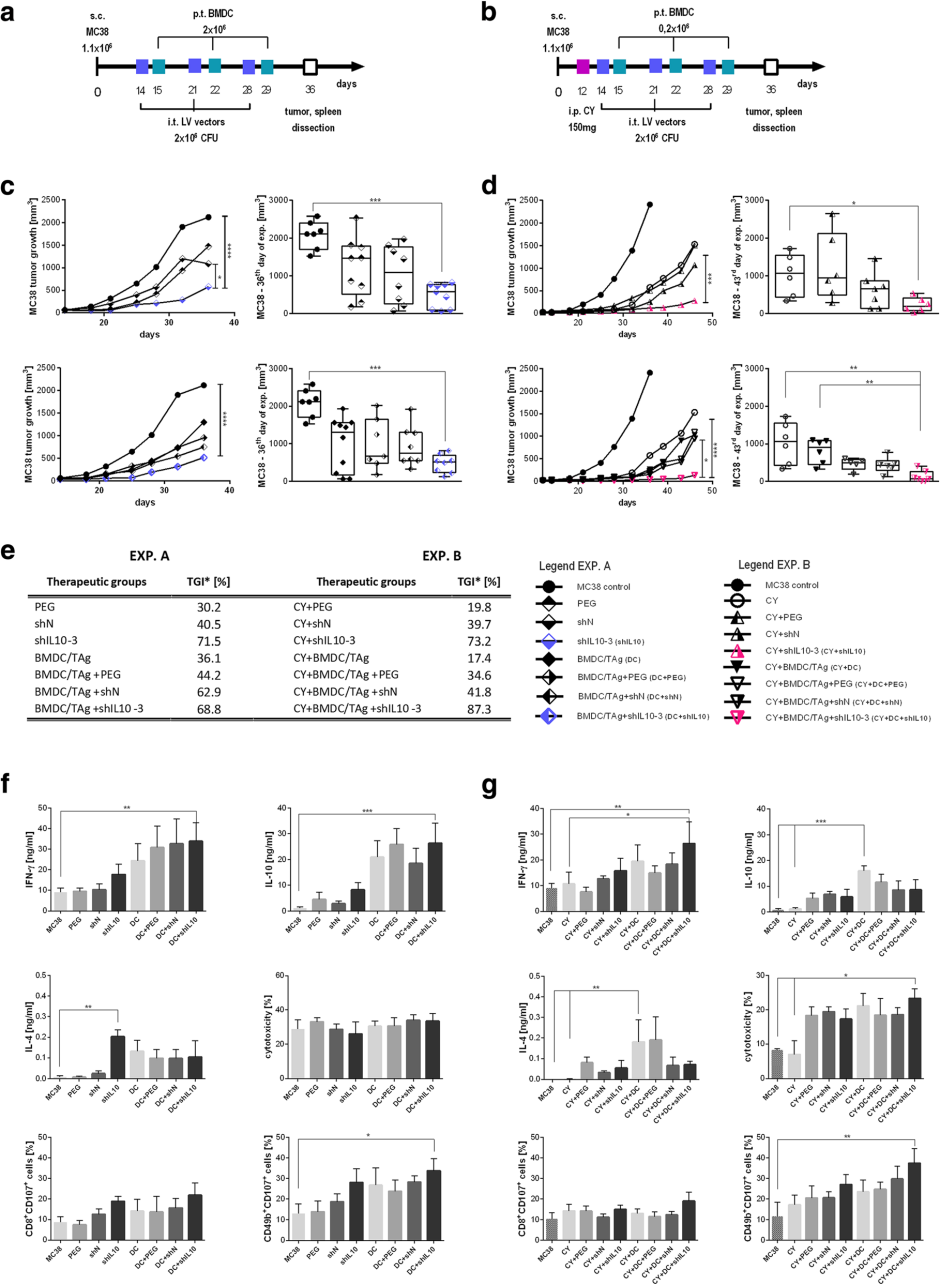
MC38 tumor growth and systemic immune response activation after immunotherapy (Exp. A) or chemoimmunotherapy (Exp. B); **a**, **b** Schemes of treatment; **c**, **d** Median of MC38 tumor growth after therapies. As a control of LV preparations, the PBS containing aggregates of polyethylene glycol (PEG) with cell debris remaining in the culture supernatant collected from untransfected Lenti-X packaging cell line was applied. The differences between groups were estimated using Friedman test. The box graphs present the median of tumor volume, calculated on the 36th day for the Exp. A and on the 43rd day for the Exp. B; **e** MC38 tumor growth inhibition (TGI) for Exp. A was calculated on the 36th day in relation to untreated group and for Exp. B - on 43rd day in relation to CY treated group; **f**, **g** Spleen cells activity in Exp. A (**f**) and Exp. B (**g**). Splenocytes obtained from the treated mice on the 36th day of experiments were restimulated in vitro in the presence of MC38 cells for 5 days. The ability of restimulated splenocytes to produce IFN-γ, IL-10 and IL-4 was determined by measurement of the cytokine concentrations in supernatants collected after 5 days of restimulation using ELISA. The presented data show the cytotoxic activity of restimulated cells towards MC38 tumor (the specific killing of DiOC18 labelled MC38 tumor cells was assessed by PI incorporation flow cytometry measurement) and the percentage of CD107a positive cells among cytotoxic CD8^+^ and CD49b^+^ cells. To calculate the mean ± SD at least 5 mice per group were analyzed in one of the two independent experiments. The differences between the groups were estimated using nonparametric Kruskal-Wallis test followed by Dunn’s multi comparison test (* *p* < 0.05, ** *p* < 0.01, *** *p* < 0.001, **** *p* < 0.0001)

The IL-10 silencing LVs were used as a component of immunotherapy based on BMDC/TAg or chemoimmunotherapy that consisted of a low dose of CY and BMDC/TAg. Schemes of therapeutic settings are presented in the Fig. 2a, b. The control groups received LVs encoding scrambled sequences of shRNA (shN) or concentrated Lenti-X culture medium (named here as PEG). The kinetics of MC38 tumor growth during the therapy, as well as tumor growth inhibition (TGI) calculated on the 36th day in the case of immunotherapy and on the 43rd day for chemoimmunotherapy are presented in the Fig. 2c, d, e. The obtained data showed that application of shIL10–3 LVs and shIL10–3 LVs in combination with BMDC/TAg induced statistically significant TGI compared to the untreated control. However, a significant difference between shIL10–3 and shN LVs was noted only when LVs were applied without BMDC (Fig. 2c, e). Moreover, in the immunotherapeutic approach the best effects were observed after application of shIL10–3 LVs. In this case, TGI calculated on the 36th day reached 71.5% in relation to untreated control. The low dose of cyclophosphamide (150 mg/kg b.w.) administered i.p. at the beginning of the therapy significantly enhanced the effectiveness of shIL10–3 LVs when applied alone and in combination with BMDC/TAg. TGI calculated on the 43rd day of experiment in relation to CY-treated control reached 73.2% or 87.3%, respectively (Fig. 2d, e). Although in the chemoimmunotherapeutic experiment the number of BMDC/TAg per injection was 10 times lower than in the immunotherapeutic approach, we clearly observed the additive effect of the DC-based vaccine, which was not visible in the group that received solely shIL10–3 LVs + BMDC/TAg.

In order to compare the effectiveness of the applied therapies in induction of systemic antitumor response the cytotoxic activity and cytokine production by spleen cells obtained from mice on the 36th day of experiment were analyzed. The presented data shows that in both therapeutic schemes, BMDC/TAg were considerably stronger inducers of IFN-γ than shIL10 LVs (Fig. 2f, g). However, their combination with shIL10–3 LVs, especially after pretreatment with CY, additionally enhanced splc’s ability to produce IFN-γ (Fig. 2g). The main difference between these two schemes was observed in Th2 type cytokine production. Namely, it was determined that production of IL-10 and IL-4 by splc from CY + shN LVs + BMDC/TAg and CY + shIL10–3 LVs + BMDC/TAg groups was considerably lower than in the group treated with CY + BMDC/TAg (Fig. 2g), whereas in the group treated with shIL10–3 LVs + BMDC/TAg the concentration of IL-10 was comparable to that obtained from the BMDC/TAg group (Fig. 2f). Changes in IL-4 production were similar to those noticed for IL-10 (Fig. 2f, g). However, in this case, shIL10–3 LVs induced higher IL-4 production than BMDC/TAg (Fig. 2f). There were no significant changes in the cytotoxic activity of the restimulated splenocytes. Nevertheless, in both schemes of treatment, we observed elevated proportions of cytotoxic NK (CD49b^+^CD107a^+^) and CTL (CD8^+^CD107a^+^) cells after treatment with shIL10 LVs when applied alone and in combination with BMDC/TAg. The effect was the most visible in the CY + shIL10–3 LVs + BMDC/TAg group (Fig. 2f, g). These results show that systemic antitumor response is activated in both schemes of treatment. However, differences in Th1 and Th2-type cytokine production observed in these two schemes of treatment suggest a noticeable shift toward Th1 type response after therapy with CY + shIL10–3 + BMDC/TAg.

### The influence of therapy composed of CY, shIL10–3 LVs and BMDC/TAg on infiltration and activity stage of immune cell subpopulations infiltrating MC38 tumors

**Fig. 3:**
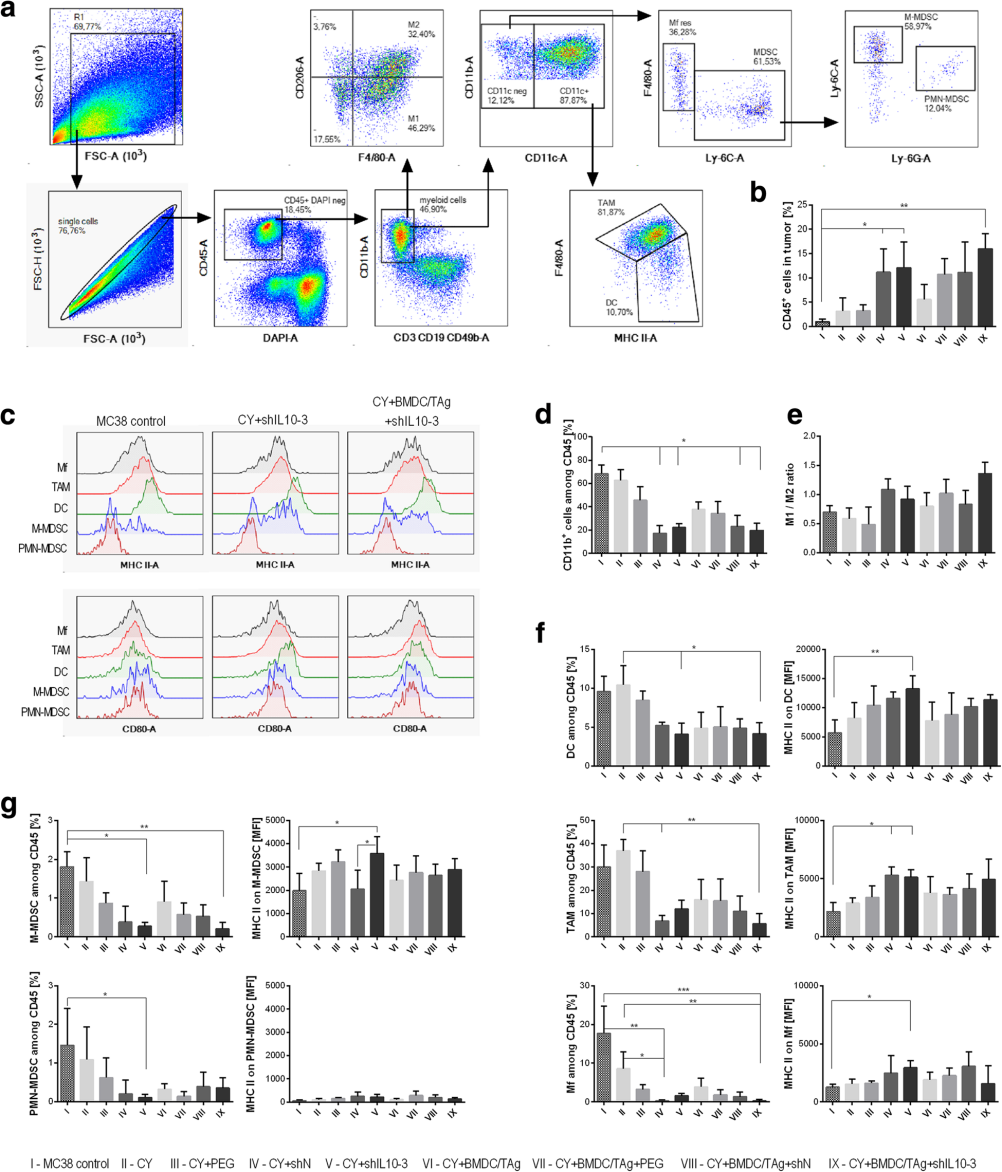
The influence of the chemoimmunotherapy on infiltration and activity stage of myeloid subpopulations infiltrating MC38 tumors. **a** The scheme of the multiparameter flow cytometric analysis showing the way of distinguishing myeloid cell subpopulations; **b** The percentage of leukocytes; **c** Representative histograms presenting changes in MHC II and CD80 expression in selected cells after treatment with shIL10–3 LVs; **d** The percentage of myeloid CD11b^+^ cells; **e** M1/M2 ratio showing changes in polarization of tumor infiltrating macrophages occurring during therapy; **f, g** subpopulations of myeloid cells and their activation stage after therapy. To calculate the mean ± SD at least 5 mice per group were analyzed. The differences between the groups were estimated using nonparametric Kruskal-Wallis test followed by Dunn’s multi comparison test (* *p* < 0.05, ** *p* < 0.01, *** *p* < 0.001)

**Fig. 4:**
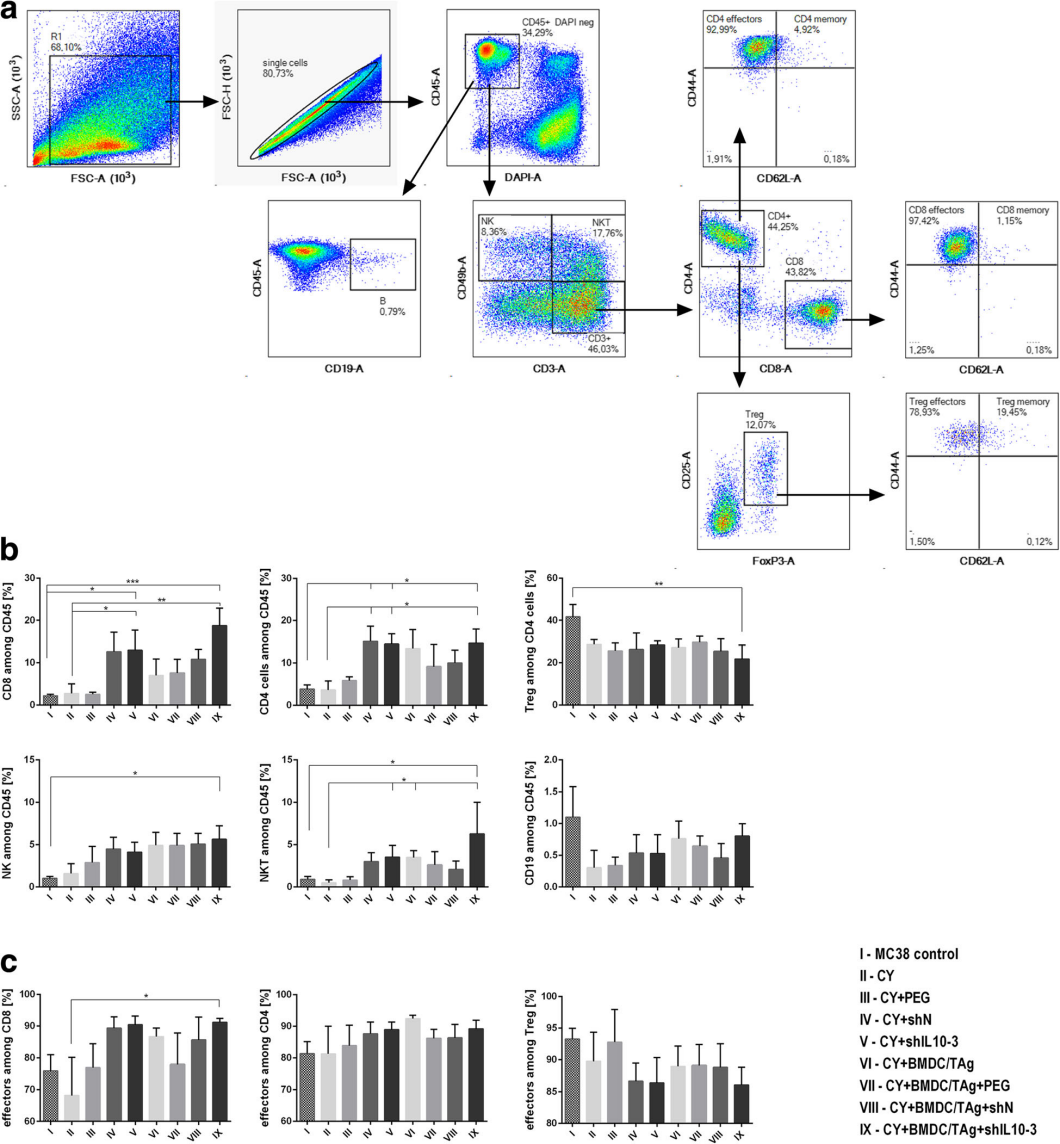
The influence of the chemoimmunotherapy on infiltration and activity stage of lymphocyte subpopulations infiltrating MC38 tumors. The figure presents scheme of multiparameter flow cytometric analysis of CTLs, Th, Treg, NK, NKT and B cells (**a**), as well as changes in their proportions after therapy (**b**). The effector cell subpopulations were distinguished on the base of CD44 and CD62L expression (**c**). To calculate the mean ± SD at least 5 mice per group were analyzed. The differences between the groups were estimated using nonparametric Kruskal-Wallis test followed by Dunn’s multi comparison test (* *p* < 0.05, ** *p* < 0.01, *** *p* < 0.001)

Special protocols for a multicolor flow-cytometric analysis were designed to determine the influence of the applied therapy on changes in immune composition of MC38 tumor microenvironment. The myeloid cell panel allowed for simultaneous identification of TAM, DCs, resident macrophages (Mf), M-MDSCs and PMN-MDSCs. Furthermore, we examined the macrophage polarization, as well as the activity stage of particular myeloid cell subpopulations basing on MHC II and CD80 expression (Fig. 3a, c). The analysis of tumors dissected on the 36th day of chemoimmunotherapy confirmed a statistically significant increase of leukocytes in the group treated with CY + shIL10–3 LVs + BMDC/TAg as compared to the untreated mice (Fig. 3b). Although the high influx of CD45^+^ cells was also observed in mice receiving CY + shIL10–3 LVs similar values were noticed for the control group receiving CY + shN LVs. In addition, we observed that both shIL10–3 and shN LVs led to significant reduction of myeloid CD11b^+^ cells in the tumor microenvironment (Fig. 3d) accompanied by decreased percentages of DCs, TAM, Mf, M-MDSCs and PMN-MDSCs (Fig. 3f, g). Nevertheless, the M1/M2 rate, which shows the influence of the applied therapy on macrophage polarization, indicates that treatment with CY + shIL10–3 LVs + BMDC/TAg caused the change of polarization of tumor infiltrating macrophages (both TAM and Mf) toward M1 (Fig. 3e). Although there were no significant changes in CD80 expression on the surface of myeloid subpopulations (Fig. 3c), it was observed that in groups treated with shIL10–3 LVs M-MDSCs and DCs were characterized by higher expression of MHC II than in other groups (Fig. 3c, f, g). This phenomenon, additionally complemented by an increased M1/M2 ratio in the CY + shIL10–3 LVs + BMDC/TAg group, indicated that the applied therapy inverted the adverse effect of TME on myeloid cells through elimination of excessive number of the cells from tumor, as well as through restoring their antitumor activity. The observed effect was accompanied by changes in proportions of tumor infiltrating lymphocytes and NK cells. The multicolor flow cytometric analysis enabled us to perform a simultaneous identification of CTLs, Th, Treg, B, NK and NKT cell subpopulations, as well as the determination of their activity stage (Fig. 4a). The analysis showed that application of the CY + shIL10–3 LVs + BMDC/TAg-based therapy induced a significant influx of CTLs, Th, NK, NKT cells and reduced the number of suppressor Treg cells in TME (Fig. 4b). Although there were no significant changes in the number of effector cells among the distinguished subpopulations of T lymphocytes, it was noted that percentages of effectors among Th and CTLs were higher than in LV treated groups as compared to the control group, while the percentage of effectors in Treg subpopulation was decreased (Fig. 4c). When we compared the TME composition after chemoimmunotherapy with that observed after shIL10–3 LV-based therapy, we noticed that shIL10–3 LVs application also induced high infiltration of effector lymphocytes and reduced number of TAM, Mf and DC in TME (Additional file 2: Figure S2). However, obtained data showed simultaneously that therapy based on shIL10–3 LVs was not able to eliminate suppressor MDSC and Treg cells from TME. To conclude, the CY + shIL10–3 LVs + BMDC/TAg therapy, in the opposite to shIL10 LVs based therapy, led to reduction of the suppressive effect caused by myeloid and Treg cells in TME, as well as induction of the effector cell influx.

### Polarization of immune response following CY, shIL10–3 LVs and BMDC/TAg treatment

**Fig. 5:**
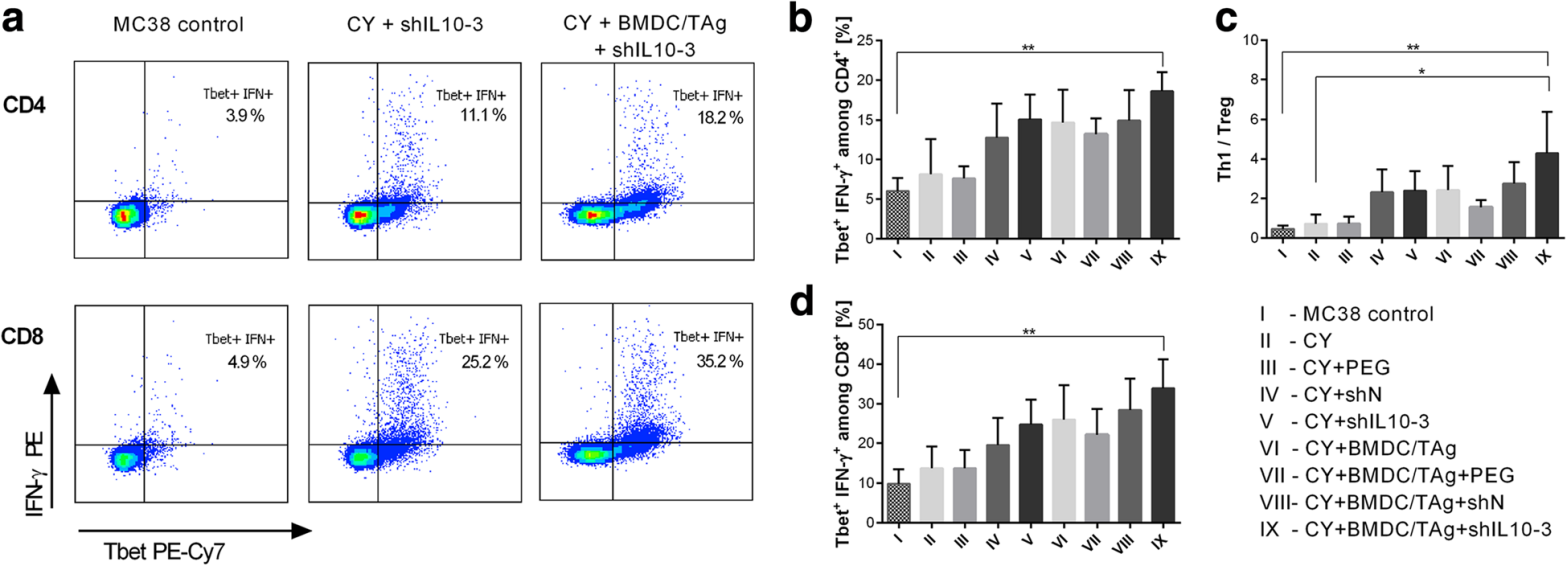
Polarization of immune response following CY, shIL10–3 LVs and BMDC/TAg treatment. **a** Representative dotplots presenting changes in the Tbet expression and IFN-γ production in CD4^+^ and CD8^+^ T lymphocytes between groups treated with shIL10–3 LVs; **b** The percentage of CD4^+^Tbet^+^IFN-γ^+^ cells; **c** The Th1 to Treg ratio; **d** The percentage of CD8^+^Tbet^+^IFN-γ^+^ cells. To calculate the mean ± SD at least 5 mice per group were analyzed. The differences between the groups were estimated using nonparametric Kruskal-Wallis test followed by Dunn’s multi comparison test (* *p* < 0.05, ** *p* < 0.01, *** *p* < 0.001)

To confirm the influence of the applied chemoimmunotherapy on tumor-derived macrophage polarization we performed functional assays, which demonstrated the induction of Th1 immune response, and thus, corroborated the shift of macrophages toward M1-type. As presented in Fig. 5, the treatment with CY + shIL10–3 LVs + BMDC/TAg induced Th1 shift with highest effectiveness. In this group, the percentage of Tbet^+^IFN-γ^+^ Th lymphocytes was considerably higher than in the control group (Fig. 5a, b). In addition, the decrease of Treg percentage after treatment with CY + shIL10–3 LVs + BMDC/TAg contributed to a significant increase in proportion of Th1 to Treg cells (Fig. 5c). The Th1 response was accompanied by high activity of CTLs. High expression of Tbet in the cells confirms increased cytotoxic activity of CTLs after treatment with CY + shIL10–3 LVs + BMDC/TAg (Fig. 5d). The obtained data indicates that the proposed combined therapy is efficient in activation of potent systemic Th1 response supporting antigen-specific CTL action.

## Discussion

The main objective of our work was to diminish the negative impact of TME-derived IL-10 on the therapeutic activity of DC-based immune- and chemoimmunotherapy. Although, different techniques of IL-10 elimination were described, the low stability of the applied molecules required frequent inoculations. Moreover, since IL-10 plays an important immunoregulatory role in the organism, it should be kept in mind that a prolonged systemic elimination of the cytokine may induce autoimmune response. In the proposed schemes of therapy, the production of IL-10 in TME was reduced locally using intratumoral (i.t.) injections of LVs encoding shRNA sequences targeting murine IL-10. We demonstrated, for the first time, the effectiveness of LV-based elimination of IL-10 from tumor nodule, its effect on the host immune response, as well as the results of its potential application in antitumor therapy.

In the first step of our study, we showed that i.t. injected shIL10–3 LVs successfully transduced cells in MC38 TME and reduced production of IL-10. This effect was accompanied by increased expression of EGFP both in tumor and immune cells isolated from tumor nodules. Previously published reports confirmed the efficiency of LVs in transduction of all cells located in TME and underlined the ability of transduced cells to accumulate in site of infection and to proliferate for even up to 3 weeks after LV inoculation [27–29]. Our data complements the previous findings by showing that the highest changes in the expression of EGFP occurred in the population of myeloid cells, while its expression in tumor cells and lymphocytes was lower. This observation may suggest that myeloid cells, which are substantial producers of IL-10 in MC38 tumor microenvironment, are main recipients of LV-derived modifications, hence shIL10 LVs could be a potent tool in regulation of TME-infiltrating myeloid cells. Further analyses revealed changes occurring in the tumor cellularity upon LV inoculation. The high number of CD8^+^ in tumor nodules on the 6th day after shN LV administration may suggest the initiation of antiviral response. This phenomenon was also described by other researchers, who showed that some of the viral proteins, which remain in lentivectors of the third generation are able to induce antiviral response [30]. Interestingly, the effect was not observed after application of LVs encoding shIL10–3 sequences. In this case, the number of CD8^+^ cells in tumors was significantly lower than in the shN LVs treated group. In this group there were no changes in the percentage of CD4^+^, NK and NKT cells. Moreover, shIL10–3 LVs transduced M-MDSCs with higher affinity than control LVs and induced a high influx of PMN-MDSCs into tumor nodules. All these data indicate that elimination of IL-10 from TME may block the immune response directed toward the LVs proteins, but simultaneously may induce MDSC-dependent immunosuppression. The controversial role of IL-10 in cancer development is still a subject of discussion. Although, some authors report its immunosuppressive effect [14, 31], others described it as an antitumor cytokine [32, 33]. Fu and co-workers, demonstrated that IL-10 is necessary for maintenance of CTLs after postclonal expansion [34]. On the other hand, Tanikawa with co-workers provided evidence showing that MDSCs from mice with *il10* knockout possess higher immunosuppressive activity than in normal mice [33]. Summarizing, blocking of IL-10 in TME can have positive effects on tumor progression. However, according to our observations, which are shared by other researchers, the anti- or protumorigenic activity of IL-10 blockers is highly context-dependent. Llopiz and co-workers showed that the application of anti-IL-10 monotherapy did not cause the expected therapeutic results, but its application with adjuvants, was capable of inducing the activation of DCs, which can potentially lead to complete B16 melanoma tumor rejection [35]. Similar observations were presented in our previous publication, where we reported the antitumor effects of anti-IL-10 Abs applied in combination with CY and DC-based vaccines used in therapy of MC38 colon carcinoma [16]. Furthermore, Kalli and co-workers showed that application of anti-IL-10 monotherapy partially inhibited the B16 melanoma and anaplastic large cell lymphoma development, while vaccination with tumor antigen stimulated DCs and anti-IL-10 Abs provided complete protection against melanoma [15]. However, due to systemic activity of anti-IL-10 Abs and risks of autoimmune response induction, the application of siRNA targeting IL-10 for ex vivo cell modification or in situ IL-10 gene silencing seems to be more attractive. Kim and co-workers utilized siRNA against IL-10 for modification of DC-based vaccines, which showed high antitumor activity when applied in immunotherapy of TC-1 tumors [36]. In our latest study, we decided to use i.t. shIL10 LV injections to gain the effect of local elimination of the cytokine. In the first presented therapeutic scheme, we applied a combination of shIL10–3 LVs with BMDC/TAg. In contrast to anti-IL-10 Abs, shIL10–3 LVs were able to induce antitumor response and caused tumor growth inhibition at the level of 71.5%. However, the shIL10–3 LVs were not able to eliminate suppressor MDSC and Treg cells from the tumor microenvironment. For this reason, there were no significant differences in tumor growth between mice treated with shIL10–3 LVs and with shIL10–3 LV + BMDC/TAg. In the second proposed scheme, we applied pretreatment with low dose of CY as an additional component of the therapy. In this case, simultaneous application of shIL10–3 LVs and BMDC/TAg significantly enhanced the TGI up to 87.3%. Comparing these two schemes of treatment, it seems that both CY and IL-10 elimination are important to improve the effectiveness of DC-based therapy. The immunomodulatory role of low doses of CY is well described in scientific literature. It can act as stimulator of effector immune cells, as well as lead to selective elimination of Tregs [37, 38]. However, as IL-10 is produced not only by Tregs but also by myeloid cells that infiltrate the MC38 tumor, the application of shIL10–3 LVs targeting mainly myeloid cells is of great importance. Moreover, the combination of cytostatics with IL-10 elimination could grant pivotal advantages. Notably, DCs in TME that is free of IL-10 are able to restore their functionality and can effectively process and present tumor antigens released from dying tumor cells [39]. In consequence of such kind of treatment the activation of a potent antitumor response is expected. Our observations indicated that, indeed, the tumor growth inhibition during therapy with CY + shIL10–3 LVs + BMDC/TAg was accompanied by significant reduction of Tregs and MDSCs number in TME and increased polarization of tumor infiltrating macrophages toward M1. The changes in myeloid cell subpopulations facilitated potent activation of local and systemic Th1-type immune response. However, since the antitumor activity of CY and DCs is well described, there are some limitations connected with in vivo application of lentiviral vectors. The approaches still face with some hurdles including efficacy of the in vivo gene delivery, necessity to use tissue-restricted promoters and immunogenicity [40]. Although, there were no adverse effects observed after LV application, we and other researchers described immunogenicity related to the components of the vectors [16, 28, 30]. Moreover, there is still a risk of insertional mutagenesis after using of the vectors. For these reasons, further vector genome engineering as well as packaging cell surface modification will likely be critical for successful application of lentiviral vectors as a fully safe, well tolerable and efficient tool for in vivo gene delivery.

## Conclusions

Concluding, the presented data indicates that reduction of IL-10 secretion in tumor microenvironment during therapy with CY and DC-based vaccines is an important and effective way to reverse the negative impact of immunosuppressive Treg and MDSC cells on peritumorally inoculated dendritic cells and to induce potent antitumor response and tumor growth inhibition. Moreover, the obtained data shows that therapeutic strategies aimed at local IL-10 elimination using lentiviral vectors should be further investigated in context of combined immuno- and chemoimmunotherapies.

## Additional files

Additional file 1: **Figure S1.** Estimation of EGFP fluorescence intensity in tumor draining lymph nodes. Tumor draining lymph nodes (tLN) were dissected on the 6th day after triple intratumoral injection of LVs encoding shIL10–3 or shN. The figure presents the intensity of EGFP fluorescence in tLN-derived myeloid cells (A) and lymphoid cells (B). To calculate the mean ± SD, at least six mice per group were analyzed. The differences between the groups were estimated using non-parametric Kruskal-Wallis test followed by Dunn’s multi comparison test (* *p* < 0.05, ** *p* < 0.01, *** *p* < 0.001, **** *p* < 0.0001). (TIF 90 kb)

Additional file 2: **Figure S2.** The influence of the shIL10–3-based therapy on lymphocyte and myeloid cell subpopulations infiltrating MC38 tumors. The figure presents changes in proportions of myeloid cells (A) and lymphoid cells (B) after therapy. To calculate the mean ± SD at least 5 mice per group were analyzed. The differences between the groups were estimated using nonparametric Kruskal-Wallis test followed by Dunn’s multi comparison test (* *p* < 0.05, ** *p* < 0.01, *** *p* < 0.001). (TIF 343 kb)

## Abbreviations

BMDCs: bone marrow-derived dendritic cells
CY: cyclophosphamide
shIL10: shRNA sequences targeting IL-10 mRNA
LVs: lentiviral vectors
MC38: murine colon carcinoma
MDSCs: myeloid derived suppressor cells
TAg: tumor antigen
