# Supplementary figures and images for: Reprogramming the murine colon cancer microenvironment using lentivectors encoding shRNA against IL-10 as a component of a potent DC-based chemoimmunotherapy
Source: J Exp Clin Cancer Res. 2018 Jun 28;37:126. doi: 10.1186/s13046-018-0799-y (PMC6025815; doi:10.1186/s13046-018-0799-y)

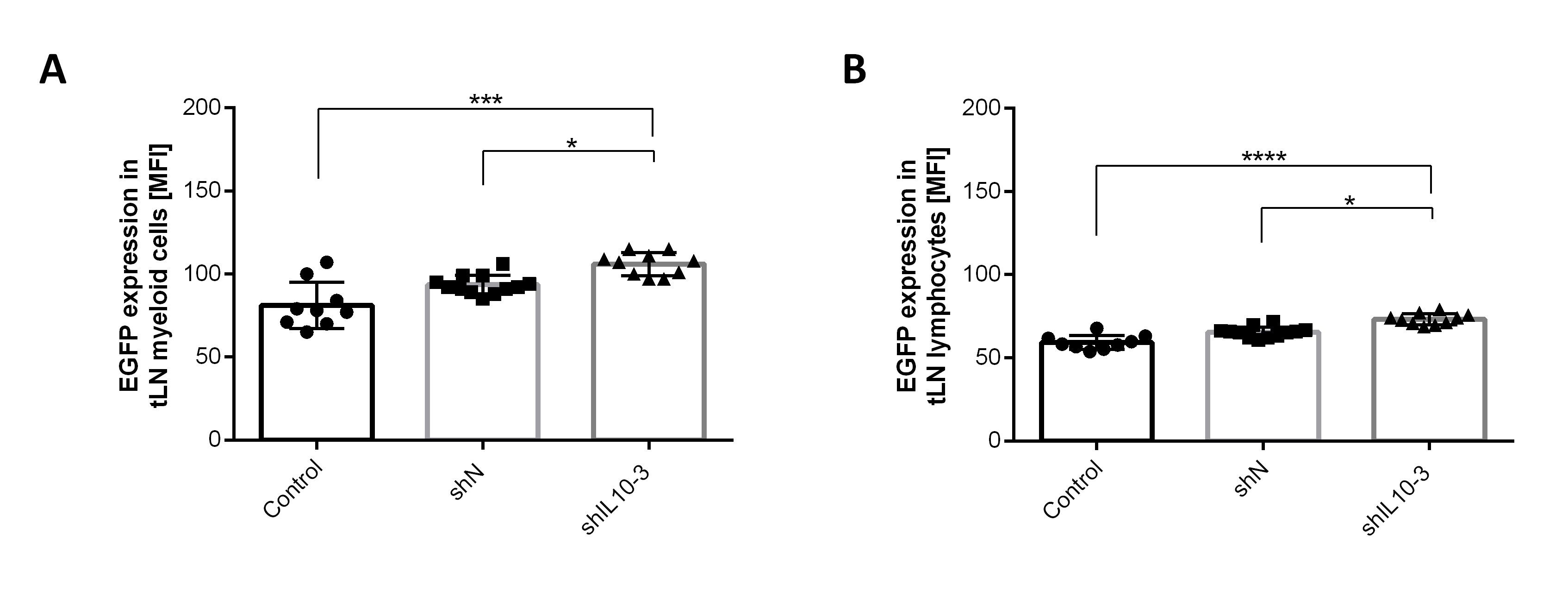

Supplement: Supplementary file 1 — Figure S1. Estimation of EGFP fluorescence intensity in tumor draining lymph nodes. Tumor draining lymph nodes (tLN) were dissected on the 6th day after triple intratumoral injection of LVs encoding shIL10–3 or shN. The figure presents the intensity of EGFP fluorescence in tLN-derived myeloid cells (A) and lymphoid cells (B). To calculate the mean ± SD, at least six mice per group were analyzed. The differences between the groups were estimated using non-parametric Kruskal-Wallis test followed by Dunn’s multi comparison test (* p < 0.05, ** p < 0.01, *** p < 0.001, **** p < 0.0001). (TIF 90 kb) [file 13046_2018_799_MOESM1_ESM.tif]

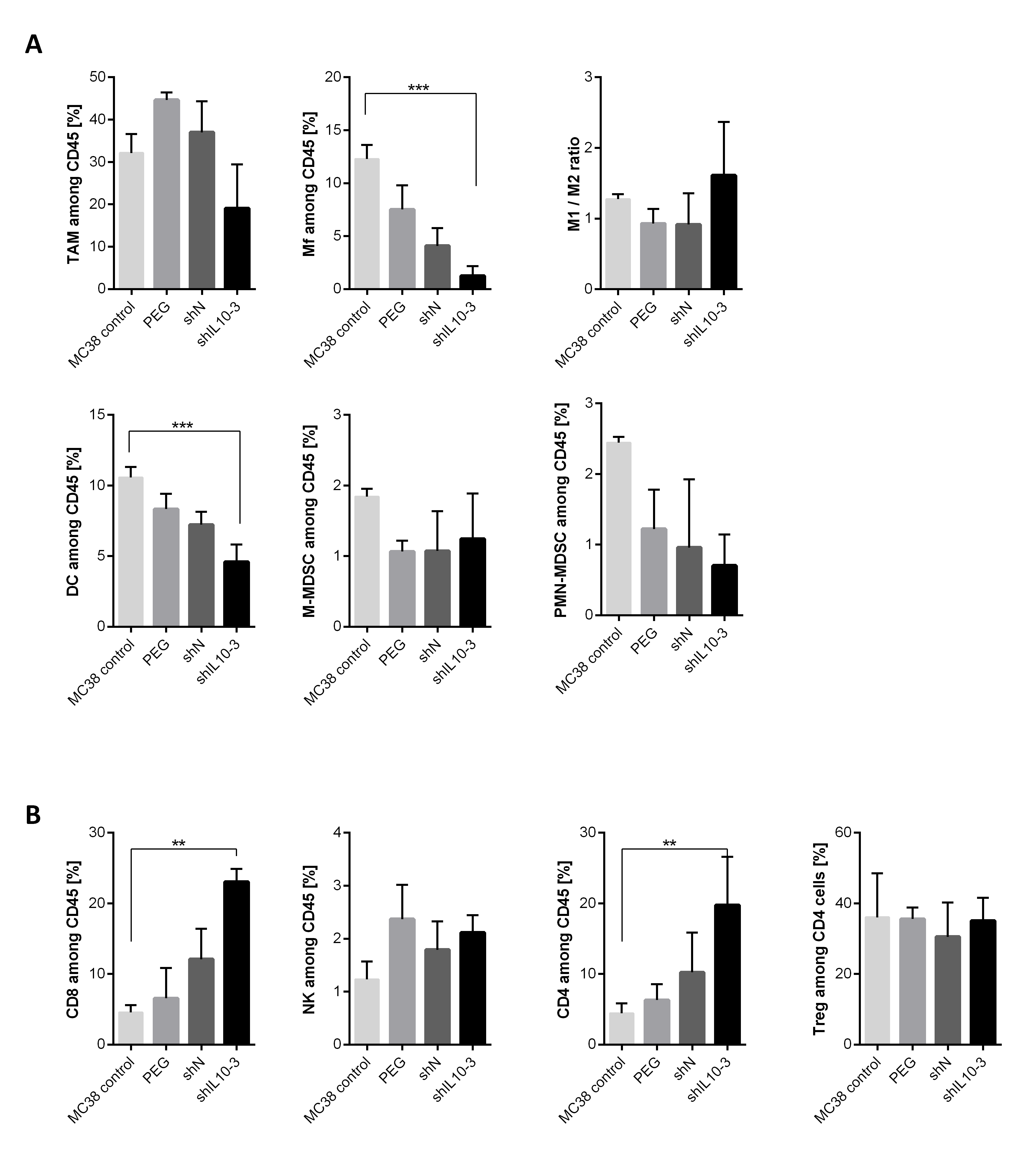

Supplement: Supplementary file 2 — Figure S2. The influence of the shIL10–3-based therapy on lymphocyte and myeloid cell subpopulations infiltrating MC38 tumors. The figure presents changes in proportions of myeloid cells (A) and lymphoid cells (B) after therapy. To calculate the mean ± SD at least 5 mice per group were analyzed. The differences between the groups were estimated using nonparametric Kruskal-Wallis test followed by Dunn’s multi comparison test (* p < 0.05, ** p < 0.01, *** p < 0.001). (TIF 343 kb) [file 13046_2018_799_MOESM2_ESM.tif]
